# Heats of Formation of Lithium Perchlorate, Ammonium Perchlorate, and Sodium Perchlorate

**DOI:** 10.6028/jres.065A.006

**Published:** 1961-02-01

**Authors:** Alexis A. Gilliland, Walter H. Johnson

## Abstract

Calorimetric measurements of the heats of solution of LiClO_4_(c), NH_4_ClO_4_(e), and NaClO_4_(c) have been made. The results have been combined with the heats of formation of KClO_4_(c), KCl(c), LiCl(c), NH_4_Cl(c), and NaCl(c), to obtain the following heats of formation:

$$\begin{array}{l} {\text{LiClO}}_{4}(c),\;\Delta \text{Hf}{}^{\circ}(25{}^{\circ}C)=-380.27\pm 1.21\;\text{kj}/\text{mole}\\ \;=-90.89\pm 0.29\;\text{kcal}/\text{mole},\\ {\text{NH}}_{4}{\text{ClO}}_{4}(c),\;=-295.98\pm 1.35\;\text{kj}/\text{mole}\\ \;=-70.74\pm 0.32\;\text{kcal}/\text{mole},\\ {\text{NaClO}}_{4}(c),\;=-382.75\pm 0.93\;\text{kj}/\text{mole}\\ \;=-91.48\pm 0.22\;\text{kcal}/\text{mole}. \end{array}$$

A brief summary of other recent data has been included.

## 1. Introduction

This investigation, a continuation of the work described in the preceding paper, was undertaken to obtain a reliable value for the heat of formation of sodium perchlorate and to provide a uniform basis for intercomparing the heats of formation of lithium, ammonium, sodium, and potassium perchlorates. Because of uncertainties in the data used for obtaining the heat of formation of perchloric acid, a method was chosen in which the heats of formation of the perchlorates were obtained in terms of the literature data on potassium perchlorate and on the corresponding chlorides.

## 2. Materials

The LiCl, NaCl, KCl, and NH_4_Cl were reagent-grade materials, dried at 130 °C, and stored in a desiccator over anhydrous magnesium perchlorate.

The KClO_4_ was reagent-grade material, recrystallized twice from water, and dried at 110 °C.

The LiClO_4_ was obtained from HEF, Inc., a corporation owned and operated jointly by the Hooker Chemical Company and the Foote Mineral Company. The following analysis in percent was furnished with the material: LiClO_4_, 99.7; H_2_O, 0.1; NaCl, 0.1; NaClO_3_, 0.005; R_2_O_3_, 0.01. However, the material had apparently been subjected to brief exposures to moisture, as it was found by the Karl Fischer method to contain approximately 1.84 percent of water. Heating the material overnight at 160 °C reduced the moisture content only slightly. However, by heating the salt to 275 °C under vacuum, the water content was reduced to not more than 0.05 percent.

The NH_4_ClO_4_ was prepared by passing gaseous ammonia into a 70-percent aqueous solution of perchloric acid; the resulting crystals were recrystallized twice from water and then dried to constant weight at 95 °C. The ammonia was obtained from the Matheson Company, who certified it to have a purity of not less than 99.99 percent. The perchloric acid was reagent-grade material which conformed with A.C.S. specifications.

The NaClO_4_ was prepared by the addition of solid reagent-grade sodium hydroxide to a 70-percent aqueous solution of perchloric acid. It was recrystallized twice from water, and dried first in air at 160 °C and then in a vacuum at 275 °C.

Each of the dried perchlorates was tested for chloride by addition of a sample to a solution of silver nitrate in nitric acid; in no case was there any clouding of the solution.

## 3. Units of Energy, Molecular Weights, and Conversion Factors

The joule was used as the unit of energy. All instruments were calibrated in terms of standards maintained at NBS. For conversion to the conventional thermo chemical calorie, one calorie is taken as 4.1840 joules.

All atomic weights were taken from the 1957 International Table of Atomic Weights [1].^1^ The heat capacities were taken, where possible, from the literature [2]. For LiClO_4_, an estimated value of 24.4 cal/deg mole was used.

## 4. Apparatus and Procedure

The glass calorimeter, thermometric system, apparatus for measurement of electrical energy, and general calorimetric procedure have been described [3,4,5]. A saturated solution of KClO_4_, consisting of approximately 0.0715 mole of KClO_4_ in 24.45 moles of water, was weighed into the calorimetric vessel, and a mixture of 0.0670 mole of KCl and 0.01 mole of KClO_4_ was added (solution I). The addition of this excess of 0.01 mole of KClO_4_ (to insure saturation of the solution) was made in all experiments. A sealed glass ampoule of LiClO_4_(c) was placed in the crushing device, the calorimeter was assembled, a platinum resistance thermometer was inserted, and the calorimeter was immersed in a thermostatically controlled water-bath maintained at 25.0 °C. The calorimeter temperature was adjusted to 24 °C by electrical heating. After an initial rating period the ampoule was broken into the solution. The calorimeter stirrer, operating at 90 rpm, provided sufficient agitation to afford thermal equilibrium in 30 min. Temperatures were observed at 1-min intervals during the reaction period and at 2-min intervals during the initial and final rating periods.

The reaction between the potassium ions and perchlorate ions caused the solution (which was already saturated) to become supersaturated with KClO_4_ and resulted in precipitation of the additional amount. The only change in the solution involved the addition of Li^+^ and a corresponding decrease in the concentration of K^+^. For an addition of 0.030 mole of LiClO_4_ the resulting solution consisted of 0.0715 mole of KClO_4_, 0.030 mole of LiCl, 0.037 mole of KCl, and 24.45 moles of water (solution II) together with 0.04 mole of solid KClO_4_.

The experiments with NH_4_ClO_4_ and with NaClO_4_ were performed in a similar manner, producing solutions IV and V respectively. To eliminate, so far as possible, the uncertainty in the state of the precipitated KClO_4_, similar experiments were run using KClO_4_. The heat measured should correspond to the transformation of dry crystalline KClO_4_ to the wet precipitated salt. The calorimeter system containing solution I was calibrated with electrical energy [4], the only change in the system being the substitution of an empty bulb for the perchlorate ampoule.

The heats of solution of KCl, LiCl, NH_4_Cl, and NaCl were determined in the same apparatus, but with a solution differing from solution I only in the quantity of KCl, which was reduced from 0.067 mole to 0.037 mole (solution III). The addition of the KCl and NH_4_Cl samples resulted in the absorption of considerable amounts of energy; to avoid corrections for the change in the concentration of the KClO_4_ with temperature, a measured quantity of electrical energy was added in each case.

A separate series of electrical-energy calibration experiments was performed, using solution III and an empty ampoule.

## 5. Results and Calculations

The results of the calibration experiments on the calorimetric system used for measurement of the heats of solution of LiClO_4_, NH_4_ClO_4_, and NaClO_4_ are given in table 1. Δ*Rc* corresponds to the corrected temperature rise of the system [6]. The energy equivalent, *E_s_*, of the “standard” system was obtained as the ratio of the quantity of electrical energy, *E*, to Δ*Rc*, the corresponding rise in temperature.

The results of the experiments on the heat of reaction of LiClO_4_ with KCl in solution I are given in table 2. Here, Δ*e* is the change in the energy equivalent from that of the “standard” system due to the heat capacity of the sample and to deviations in the mass of the glass bulbs from that of the reference bulb, (0.444g). The energy evolved, *q*, was obtained as the product of Δ*Rc* and the energy equivalent of the actual calorimetric system, *E_s_+* Δ*e*.

The results of the experiments on the heats of reaction of NH_4_ClO_4_ and NaClO_4_ with KCl in solution I are given in tables 3 and 4, respectively. The results of the experiments on the heat of addition of KClO_4_ to solution I are given in table 5.

The results of the calibration experiments for the system containing solution III are given in table 6. The results of the experiments on the heats of solution of LiCl, KCl, NH_4_Cl, and NaCl in solution III are given in table 7, 8, 9, and 10, respectively.

The concentrations of the calorimetric solutions involved, on a molar basis, are as follows:

$$\begin{array}{l} {}[2.38\;{\text{KClO}}_{4}+2.23\;\text{KCl}+815\;H_{2}O]\;\text{solution I},\\ {}[2.38\;{\text{KClO}}_{4}+1.23\;\text{KCl}+\text{LiCl}+815\;H_{2}O]\;\text{solution II},\\ {}[2.38\;{\text{KClO}}_{4}+1.23\;\text{KCl}+815\;H_{2}O]\;\text{solution III},\\ {}[2.38\;{\text{KClO}}_{4}+1.23\;\text{KCl}+{\text{NH}}_{4}\text{Cl}+815\;H_{2}O]\;\text{solution IV},\\ {}[2.38\;{\text{KClO}}_{4}+1.23\;\text{KCl}+\text{NaCl}+815\;H_{2}O]\;\text{solution V}. \end{array}$$

The calorimetric processes and the corresponding changes in enthalpy are:

$$\begin{array}{l} {\text{LiClO}}_{4}(c)+[I]=[\text{II}]+{\text{KClO}}_{4}(\text{pptd}),\\ \Delta H(25\;{}^{\circ}C)=-62.839\pm 0.216\;\text{kj}/\text{mole}, \end{array}$$ (1)

$$\begin{array}{l} {\text{NH}}_{4}{\text{ClO}}_{4}(c)+[I]=[\text{IV}]+{\text{KClO}}_{4}(\text{pptd}),\\ \Delta H(25\;{}^{\circ}C)=-0.252\pm 0.144\;\text{kj}/\text{mole}, \end{array}$$ (2)

$$\begin{array}{l} {\text{NaClO}}_{4}(c)+[I]=[V]+{\text{KClO}}_{4}(\text{pptd}),\\ \Delta H(25\;{}^{\circ}C)=-21.166\pm 0.202\;\text{kj}/\text{mole}, \end{array}$$ (3)

$$\begin{array}{l} \text{KCl}(c)+[\text{III}]=[I],\\ \Delta H(25\;{}^{\circ}C)=3.850\pm 0.112\;\text{kj}/\text{mole}, \end{array}$$ (4)

$$\begin{array}{l} \text{LiCl}(c)+[\text{III}]=[\text{II}],\\ \Delta H(25\;{}^{\circ}C)=-34.431\pm 0.096\;\text{kj}/\text{mole}, \end{array}$$ (5)

$$\begin{array}{l} {\text{NH}}_{4}\text{Cl}(c)+[\text{III}]=[\text{IV}],\\ \Delta H(25\;{}^{\circ}C)=19.078\pm 0.062\;\text{kj}/\text{mole}, \end{array}$$ (6)

$$\begin{array}{l} \text{NaCl}(c)+[\text{III}]=[V],\\ \Delta H(25\;{}^{\circ}C)=7.002\pm 0.128\;\text{kj}/\text{mole}, \end{array}$$ (7)

$$\begin{array}{l} {\text{KClO}}_{4}(c)+[I]=[I]+{\text{KClO}}_{4}\;(\text{pptd}),\\ \Delta H(25\;{}^{\circ}C)=-0.075\pm 0.030\;\text{kj}/\text{mole}. \end{array}$$ (8)

The appropriate combinations of the above equations yield the following processes:

$$\begin{array}{l} {\text{LiClO}}_{4}(c)+\text{KCl}(c)=\text{LiCl}(c)+{\text{KClO}}_{4}(c),\\ \Delta H{}^{\circ}(25\;{}^{\circ}C)=-24.483\pm 0.260\;\text{kj}/\text{mole},\\ \;=-5.855\pm 0.062\;\text{kcal}/\text{mole}, \end{array}$$ (9)

$$\begin{array}{l} {\text{NH}}_{4}{\text{ClO}}_{4}(c)+\text{KCl}(c)={\text{NH}}_{4}\text{Cl}(c)+{\text{KClO}}_{4}(c),\\ \Delta H{}^{\circ}(25\;{}^{\circ}C)=-15.405\pm 0.195\;\text{kj}/\text{mole},\\ \;=-3.682\pm 0.047\;\text{kcal}/\text{mole}, \end{array}$$ (10)

$$\begin{array}{l} {\text{NaClO}}_{4}(c)+\text{KCl}(c)=\text{NaCl}(c)+{\text{KClO}}_{4}(c),\\ \Delta H{}^{\circ}(25\;{}^{\circ}C)=-24.243\pm 0.266\;\text{kj}/\text{mole},\\ \;=-5.794\pm 0.064\;\text{kcal}/\text{mole}. \end{array}$$ (11)

We have combined the results given in eqs 9, 10, and 11 with our value of −4.02 ±0.40 kj/mole for the heat of decomposition of potassium perchlorate, reported in the preceding paper [7], and with values for the heats of formation of KCl(c), LiCl(c), NH_4_Cl(c), and NaCl(c) [2], and have obtained the following heats of formation:

$$\begin{array}{l} {\text{LiClO}}_{4}(c),\;\Delta \text{Hf}{}^{\circ}(25{}^{\circ}C)=-380.27\pm 1.21\;\text{kj}/\text{mole}\\ \;=-90.89\pm 0.29\;\text{kcal}/\text{mole},\\ {\text{NH}}_{4}{\text{ClO}}_{4}(c),\;\Delta \text{Hf}{}^{\circ}(25{}^{\circ}C)\;=-295.98\pm 1.35\;\text{kj}/\text{mole}\\ \;=-70.74\pm 0.32\;\text{kcal}/\text{mole},\\ {\text{NaClO}}_{4}(c),\;\Delta \text{Hf}{}^{\circ}(25{}^{\circ}C)=-382.75\pm 0.93\;\text{kj}/\text{mole}\\ \;=-91.48\pm 0.22\;\text{kcal}/\text{mole}. \end{array}$$

## 6. Discussion

Rossini, Wagman, Evans, Levine, and Jaffe [2] have selected −69.42 and −92.18 kcal/mole, respectively, for the standard heats of formation of NH_4_ClO_4_(c) and NaClO_4_(c), based upon the data prior to 1950. However, since these values were based on −103.6 kcal/mole for the heat of formation of KClO_4_, we have made a correction of 0.38 kcal/mole and have obtained −69.04 and −91.80 kcal/mole, respectively, for NH_4_ClO_4_(c) and NaClO_4_(c).

Markowitz, Harris, and Stewart [8] measured the heats of reaction between aqueous solutions of LiOH and HClO_4_, and the heat of solution, in water, of anhydrous LiClO_4_. They obtained −91.70 kcal/mole for the heat of formation of LiClO_4_ based on the heats of formation of LiOH(aq) and HClO_4_(aq) [2]. However, since the heat of formation of HClO_4_(aq) was based on −103.6 kcal/mole for the heat of formation of KClO_4_ [2], we have made a correction of 0.38 kcal to their data, obtaining −91.32 kcal/mole for the heat of formation of LiClO_4_(c).

Birky and Hepler [9] measured the heats of solution in water, of KClO_4_(c), NH_4_ClO_4_(c), and LiClO_4_(c), and obtained −70.63 and −91.11 kcal/mole, respectively, for the heats of formation of NH_4_ClO_4_(c) and LiClO_4_(c). Their data are based on the heat of formation of KClO_4_(c) [7] and on the heats of formation of K^+^(aq), Li^+^(aq), and NH_4_^+^(aq) [2].

The heats of formation in kcal/mole at 25 °C obtained by the various investigators are compared with the results of the present investigation in the following tabulation:

**Table:** 

	LiClO_4_	NH_4_ClO_4_	NaClO_4_
			
Rossini, Wagman, Evans, et al. [2].	…………	−69.04	−91.80
Markowitz, Harris, and Stewart [8].	−91.32	…………	…………
Birky and Hepler [9]	−91.11	−70. 63	…………
This investigation	−90.89±0.29	−70.74±0.32	−91.48±0.22

## Figures and Tables

**Table 1:** Results of the calibration experiments with solution I

Experiment No.	Δ*Rc*	*E*	*E_s_*
			
	*ohm*	*j*	*j/ohm*
1	0.101111	2202.00	21778.0
2	.101065	2200.45	21772.6
3	.100631	2190.57	21768.3
4	.060589	1319.84	21783.5
5	.082227	1791.93	21792.5
6	.076776	1671.91	21776.5
	
Mean			21778.6
Standard deviation of the mean			±3.5

**Table 2:** Heat reaction of *LiClO*_4_ with *KCl* in solution I

Experiment No.	Δ*Rc*	Δ*e*	*q*	LiClO_4_	−ΔH(25 °C)
					
	*ohm*	*j/ohm*	*j*	*mole*	*kj/mole*
1	0.074983	27.1	1635.1	0.026135	62.564
2	.074319	29.3	1620.7	.025660	63.161
3	.082012	29.8	1788.6	.028523	62.707
4	.078088	27.6	1702.8	.027131	62.762
5	.072577	27.0	1582.6	.025120	63.002
	
Mean					62.839
Standard deviation of the mean					±0.108

**Table 3:** Heat of reaction of *NH*_4_*ClO*_4_ with *KCl* in solution I

Experiment No.	Δ*Rc*	Δ*e*	*q*	NH_4_ClO_4_	−ΔH(25 °C)
					
	*ohm*	*j/ohm*	*j*	*mole*	*kj/mole*
1	0.000472	43.8	10.30	0.024314	0.424
2	.000250	38.4	5.45	.021541	.253
3	.000355	29.0	7.75	.021697	.357
4	.000430	41.4	9.38	.023137	.405
5	.000034	47.9	0.74	.026663	.028
6	.000066	54.4	1.45	.030132	.048
	
Mean					0.252
Standard deviation of the mean					±0.072

**Table 4:** Heat of solution of *NaClO*_4_ with *KCl* in solution I

Experiment No.	Δ*Rc*	Δ*e*	*q*	NaClO_4_	−ΔH(25 °C)
					
	*ohm*	*j/ohm*	*j*	*mole*	*kj/mole*
1	0.026518	29.7	578.31	0.027532	21.005
2	.024535	27.0	535.00	.025622	20.880
3	.026419	28.4	576.12	.027122	21.242
4	.024436	26.5	532.83	.025080	21.245
5	.023510	24.5	512.59	.023889	21.457
	
Mean					21.166
Standard deviation of the mean					±0.101

**Table 5:** Heat of addition of *KCl*O_4_ to solution I

Experiment No.	Δ*Rc*	Δ*e*	*q*	KClO_4_	−ΔH(25 °C)
					
	*ohm*	*j/ohm*	*j*	*mole*	*kj/mole*
1	0.000108	21.8	2.35	0.01991	0.118
2	.000071	24.7	1.55	.02225	.070
3	.000034	25.3	0.74	.02287	.032
4	.000107	27.9	2.33	.02424	.096
5	.000006	25.4	0.13	.02240	.058
	
Mean					0.075
Standard deviation of the mean					±0.015

**Table 6:** Results of the calibration experiments with solution III

Experiment No.	Δ*Rc*	*E*	*E_s_*
			
	*ohm*	*j*	*j/ohm*
1	0.100665	2203.51	21889.5
2	.101224	2215.17	21883.8
3	.100624	2200.52	21868.7
4	.085369	1869.01	21893.3
5	.099602	2179.06	21877.7
6	.099575	2177.48	21867.7
	
Mean			21880.1
Standard deviation of the mean			±4.3

**Table 7:** Heat of solution of *LiCl* in solution III

Experiment No.	Δ*Rc*	Δ*e*	*q*	LiCl	−ΔH(25 °C)
					
	*ohm*	*j/ohm*	*j*	*mole*	*kj/mole*
1	0.059686	17.8	1307.0	0.0378442	34.536
2	.057614	17.6	1261.6	.0368166	34.267
3	.085069	26.6	1863.6	.0539895	34.518
4	.071628	22.6	1568.8	.0456099	34.397
5	.078054	24.9	1709.8	.0496536	34.435
	
Mean					34.431
Standard deviation of the mean					±0.048

**Table 8:** Heat of solution of *KC1* in solution III

Experiment No.	Δ*Rc*	Δ*e*	*E*	*q*	KCl	−ΔH(25 °C)
						
	*ohm*	*j/ohm*	*j*	*j*	*mole*	*kj/mole*
1	0.099847	14.4	2293.8	−107.7	0.029285	−3.678
2	.099712	13.7	2282.6	−99.5	.026790	−3.714
3	.098928	14.0	2279.7	−113.8	.028950	−3.931
4	.100757	15.8	2324.1	−117.9	.031030	−3.800
5	.099508	15.1	2297.4	−118.7	.029810	−3.982
6	.099260	14.5	2292.9	−119.6	.029946	−3.994
	
Mean						−3.850
Standard deviation of the mean						±0.056

**Table 9:** Heat of solution of *NH*_4_*Cl* in solution III

Experiment No.	Δ*Rc*	Δ*e*	*E*	*q*	NH_4_Cl	−ΔH(25 °C)
						
	*ohm*	*j/ohm*	*j*	*j*	*mole*	*kj/mole*
1	0.089184	31.1	2673.1	−719.0	0.037639	−19.103
2	.085337	31.0	2567.2	−697.4	.036725	−18.990
3	.090007	29.0	2629.4	−657.4	.034397	−19.112
4	.085593	28.1	2527.3	−652.1	.034031	−19.162
5	.089177	34.3	2720.9	−766.6	.040300	−19.022
	
Mean						−19.078
Standard deviation of the mean						±0.031

**Table 10:** Heat of solution of *NaCl* in solution III

Experiment No.	Δ*Rc*	Δ*e*	*q*	NaCl	−ΔH(25 °C)
					
	*ohm*	*j/ohm*	*j*	*mole*	*kj/mole*
1	−0.009690	15.6	−212.17	0.029920	−7.091
2	−.009086	13.8	−198.93	.028985	−6.863
3	−.009335	15.9	−204.40	.029964	−6.822
4	−.009489	14.9	−207.76	.028691	−7.241
5	−.009642	14.6	−211.11	.029950	−7.049
6	−.009153	14.7	−200.40	.028847	−6.947
	
Mean					−7.002
Standard deviation of the mean					±0.064

## Footnotes

1 Figures in brackets indicate the literature references at the end of this paper.
